# Assessing Barriers to Primary Prevention of Cardiovascular Diseases in Low and Middle-Income Countries: A Systematic Review

**DOI:** 10.7759/cureus.65516

**Published:** 2024-07-27

**Authors:** Nathan Mulure, Hewad Hewadmal, Zahid Khan

**Affiliations:** 1 Public Health, Kenya Medical Research Institute (KEMRI), Nairobi, KEN; 2 Internal Medicine, Desert Valley Hospital, Victorville, USA; 3 Acute Medicine, Mid and South Essex NHS Foundation Trust, Southend-on-Sea, GBR; 4 Cardiology, Barts Heart Centre, London, GBR; 5 Cardiology and General Medicine, Barking, Havering and Redbridge University Hospitals NHS Trust, London, GBR; 6 Cardiology, Royal Free Hospital, London, GBR; 7 Preventive Cardiology, University of South Wales, London, GBR; 8 Cardiology, University of Buckingham, London, GBR

**Keywords:** absence of shared decision-making, absence of national guidelines, physician barriers, health financing barriers, cambodia and mongolia steps survey analysis, inadequate information from health care providers, patient-reported barriers, major adverse cardiac events (mace), low and middle-income countries (lmics), barriers to primary prevention of cardiovascular diseases

## Abstract

The incidence of cardiovascular diseases (CVDs) in low- and middle-income countries (LMICs) has greatly increased. Previously dominated by infectious diseases, LMICs are the new epicentre of CVDs. CVD is a common problem amongst the population in the African continent; however, many countries in LMICs lack the resources to stem the rise of CVDs.

A systematic review was conducted between March and July 2023 to assess barriers to the primary prevention of CVDs in studies conducted in LMICs. Online databases, such as Embase, Cochrane, Scopus, and MEDLINE, were consulted. Keywords included primary prevention, cardiovascular diseases, diabetes, weight loss, and physical fitness, all of which focused on LMICs. To enrich the literature review, efforts were made to check other listed references and more papers were retrieved. The inclusion criteria were countries in LMICs, CVD, full-text, and peer-reviewed journals. Qualitative and quantitative studies were included. Exclusion criteria included high-income countries, secondary prevention, and research unrelated to CVDs, such as barriers in oncology or mental health.

A total of 1089 papers were retrieved from the search engines. After applying the exclusion criteria for LMICs, only 186 papers were retained. A further search for quality, relevance, and duplicity reduced the qualifying number to 50 papers. Further efforts to retrieve the data and examine the quality of the studies resulted in 18 final selected studies. Three categories emerged based on the type of barriers: physician barriers, patient barriers, and health system barriers. Evidently, in several LMICs, guidelines for CVD prevention were lacking, and too much emphasis was placed on secondary prevention at the expense of primary prevention, a lack of human resources, and inadequate infrastructure. Overworked healthcare providers were unable to allocate adequate time to the patients. There was no shared decision-making process. Patient barriers included lack of motivation, no symptoms, low level of education, no insurance, long physical distances to the facilities, and inadequate medication or stock out. Some of the major barriers included closing and opening hours, poor operating space, inadequate funding from the government or donors, and lack of electronic medical services.

There are many barriers to accessing primary prevention services for CVDs. These barriers can be divided into patient, physician, or health system barriers. More research needs to be conducted in LMICs to address the increasing risk factors for CVDs. Greater investment is required by national governments to provide more resources. Task shifting and shared decision-making are some of the quick wins.

## Introduction and background

This review seeks to understand the barriers to the primary prevention of cardiovascular diseases (CVDs) in low- and middle-income countries (LMICs). Accessing health care has been debated for several years. The incidence of CVDs has increased by over 50% in Sub-Saharan Africa over the past 30 years, accounting for almost 38% of non-communicable diseases in Africa [1,2]. Access was first described by the Institute of Medicine (IOM), which stated that it is the “timely use of personal health services to achieve the best outcome”. Access to health services has four main qualities: coverage, services, timeliness, and workforce [3]. These are important in ensuring universal health coverage (UHC), which, according to the World Bank, is to “ensure that people have access to health care that they need without suffering financial hardship” [3]. The World Bank further qualifies UHC to include the entire continuum of care, including health promotion, disease prevention, treatment, rehabilitation, and palliative care. Levesque et al. developed a framework and conceptualized five dimensions of accessibility, including approachability, acceptability, availability and accommodation, affordability, and appropriateness. In this framework, the abilities of five corresponding populations interact with the dimensions of accessibility to generate access. The five dimensions of abilities include the ability to perceive, seek, reach, pay, and engage [3].

A patient’s search for actual utilization of care could include a delay in seeking health care and a delay between the desire for care and the actual search for care [3]. Services available to patients must be acceptable, considering religious and cultural practices. They also need to be within reach with appropriate opening and closing hours. Affordability is key, especially in LMICs, where more than 40% of healthcare expenses are out-of-pocket [4]. On the other hand, the patient must possess key empowering attributes such as health literacy, mobility, social support, and health insurance, as well as information provided by the healthcare worker to empower the patient with appropriate information [3]. CVDs are becoming increasingly important in LMICs. According to the World Bank, LMICs are mainly found in Africa, Latin America, the Caribbean, the Middle East, and South Asia [5]. According to the new World Bank classification, which came into effect in July 2021, low-income countries have a gross national income (GNI) of <1,045, while lower-middle-income countries range from 1,046 to 4,095 United States dollars (USD) each year (Table 1).

**Table 1:** Country classification based on gross national income per capita. Reference [5].

Group	July 1, 2021 (new)	July 1, 2020 (old)
Low income	<1,045	<1,035
Lower-middle income	1,046-4,095	1,035-4,045
Upper-middle income	4,096-12,695	4,046-12,535
High income	>12,695	>12,535

The number of low and lower-middle-income countries is estimated to be about 132. CVDs have been identified as a leading course of mortality and morbidity in many parts of LMICs [1]. CVD includes several diseases affecting the arteries, such as coronary artery diseases (CADs), nerves to the blood vessels, rheumatic heart diseases, and venous thromboembolism [6]. It is estimated that CVDs account for over 80% of global mortality and 88% come from LMICs [7].

Primary prevention of CVDs aims to avoid major adverse cardiac events (MACEs) such as stroke and myocardial infarction, hence reducing early morbidity or mortality [8]. According to Liu et al., the Coronary Artery Risk Development in Young Adults (CARDIA) study amongst young adults demonstrated 80% CVD risk reduction in patients by avoiding high-risk behaviour [9]. In the UK, CVD accounts for about 34% of mortality, and the number is slightly higher in the European Union (EU) at 40% [6]. This is despite all the advancements and good quality care in Western countries. In India, CVD has been estimated to contribute to more than 28% of total deaths and 14% of disability-adjusted years. In India, the consequences of CVD appear 10 years earlier than in the Western world, progressing rapidly with a high mortality rate [10]. By 2009, the economic burden of CVD in the EU was about 109 billion United States dollars (USD), which was about 9% of the health budget in the union [11].

The primary preventive efforts to reduce CVD are not optimal and there are several barriers and gaps to attaining optimal prevention [12]. According to the WHO, primary prevention of CVD is key to reducing morbidity and mortality. Lifestyle changes are important in the primary prevention of CVD [13]. Lack of resources or government prioritization has been identified as barriers to the effective primary preventative strategy in LMICs [14].

Risk scoring is an important tool in categorizing a population’s risk. Scores have been used in higher-income countries for many years, especially in the USA and Western Europe [15]. It is not easy to generalize the use of these risk scores to other populations [16]. In LMICs, risk scores are yet to be developed, and physicians rely on Western risk scores to manage patients in the local context. Evidence from LMICs is either lacking or has not been incorporated in generating risk scores [17].

Smoking is a major predictor of CVD with a significantly higher all-cause mortality in smokers. According to the WHO, smoking accounts for about 10% of CVD mortality [18]. Tobacco usage causes about 7 million deaths yearly due to CVD complications and is associated with thrombotic events leading to ischaemia and infarctions [18,19]. Second-hand smoke negatively affects the victims and could lead to CVD complications. Despite overwhelming evidence of the benefits of smoking cessation and its positive impact on population health, there are few services for smoking cessation in LMICs and only 15% of the world population has access to these services [18]. The skills gap could be bridged by offering more targeted training to medical students [20]. Importantly, medical students should be discouraged from taking up cigarette smoking, as this often develops during medical school training [21].

Physical activity and exercise possess cardiovascular benefits [13]. Research shows that physically active individuals have 50% lower incidences of CADs compared to sedentary subjects [22]. The prevalence of obesity in Western countries has been documented to be as high as 25%. It is an independent and modifiable risk factor that is associated with comorbidities and cardiovascular mortality [23].

In the LMICs, comprising mostly Africans and Asians, a BMI of 27.5 kg/m2 or above is considered obese due to the predisposition to central obesity amongst these populations [24]. Despite these well-documented methods of preventing CVDs, LMICs are still playing catch up with the more developed countries, as far as preventative medicine is concerned.

## Review

Aim of the study

The aim of the current study included identifying factors that hinder the early diagnosis of CVDs in LMICs, establishing factors that prohibit access to early and continuous treatment of CVDs, and the practicality of lifestyle changes, nutrition, and exercise in preventing CVDs; and identifying geographical and ethnic differences in the prevention of CVDs and identifying the barriers to primary prevention of CVDs in LMICs. The inclusion criteria were studies published in the English language only, qualitative or quantitative studies, studies conducted in low-income countries (LICs) or LMICs as per the World Bank's definition, studies relating to non-communicable diseases (NCDs), and studies with full-text peer-reviewed articles. The exclusion criteria included studies done before 2000 and after 2023, non-English Language articles, studies with poor or ill-defined methodologies, studies lacking statistical analysis, and studies from high-income countries (HICs).

Methodology

A literature search was conducted with keywords including barriers, access, primary prevention, and cardiovascular diseases. The keywords used included “primary prevention in low and middle-income countries”, “primary prevention in middle-income countries”, “primary prevention barriers in low-income countries”, “patient barriers to primary cardiovascular prevention in the African continent”, “healthcare staff shortages in low and middle-income countries”, “shared decision-making in low and middle-income countries”, “healthcare facilities in low and middle-income countries”, “lack of healthcare resources in low and middle-income countries”, “integrated healthcare system in low and middle-income countries”, “barriers to healthcare facilities in low and middle-income countries”, “cardiovascular prevention in low and middle-income countries and barriers”, “barriers to healthcare and primary prevention in Africa, Asia, Middle-East, Asia Pacific, Southeast Asia, Central Asia and South America”, and “cardiovascular risk reduction in low and middle-income countries”. We used a combination of these keywords for literature search across various search engines. The Author also searched specific elements of preventive CVDs, such as barriers to smoking cessation in LMICs, barriers to weight in LMICs, and barriers to access to medicines in LMICs. Databases included PubMed, Embase, and Scopus. Google Scholar and Find It search engines were also utilized. Another search strategy was conducted on preventative measures and their respective barriers, for example, “barriers to smoke cessation”, “low middle income”, “barriers to weight loss programs low middle income”, and “barriers to access to cardiovascular drugs primary prevention low middle income”, yielding a few additional papers.

**Figure 1:**
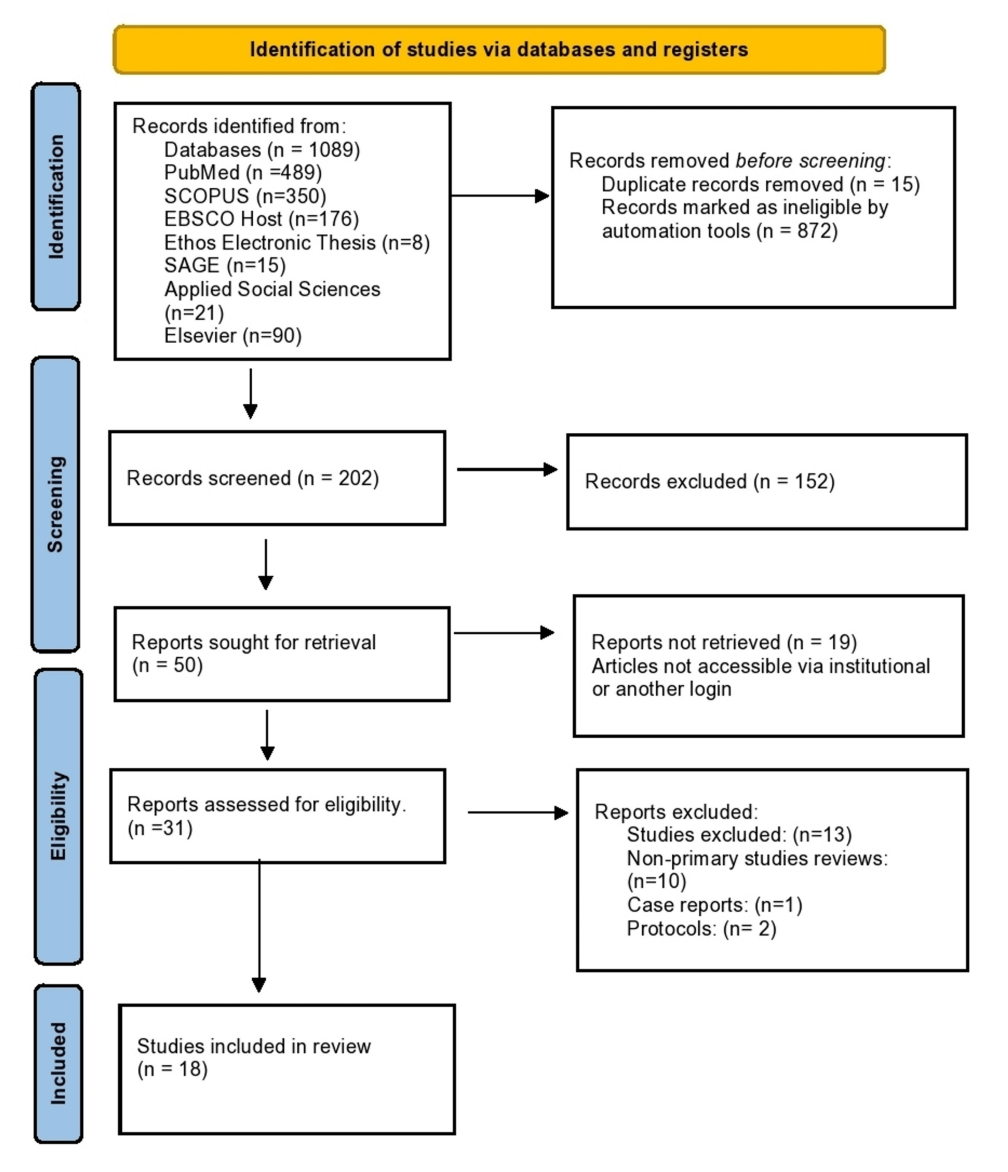
Preferred Reporting Items for Systematic Reviews and Meta-Analyses (PRISMA) flow diagram.

The initial search using several databases yielded a total of 1089 papers. Studies that did not meet the inclusion criteria were removed from the list of included studies. All reviews that did not utilize primary patient data were excluded. Two reviews screened were large multi-country studies and had access to individual data from national surveys. Individual papers were also screened, and duplicates were removed from the final analysis. A total of 872 studies did not meet the inclusion criteria after reviewing their titles and were removed from the list. A further 15 studies were removed due to being duplicates. From the remaining 186 studies, a further 136 studies were excluded after screening their abstracts, as shown in the Preferred Reporting Items for Systematic Reviews and Meta-Analyses (PRISMA) flow diagram (Figure 1). Most of these studies were from HICs that did not focus on CVDs and focused on other health conditions or parameters such as cancer, availability of surgical treatment, and respiratory diseases. Approximately, 80% of the studies were excluded based on geographical region. We were able to retrieve only 31 full-text articles from the 50 articles and nine studies were excluded for being case reports, non-primary studies, or study protocols only. Nigeria, Sierra Leone, and Thailand were some of the countries that had reported primary research on barriers to accessing primary prevention of CVDs. Both qualitative and quantitative studies were assessed for soundness in methodology and data quality. The number of participants, sample size calculations, and the presence or absence of bias were considered.

Risk of bias assessment

**Figure 2:**
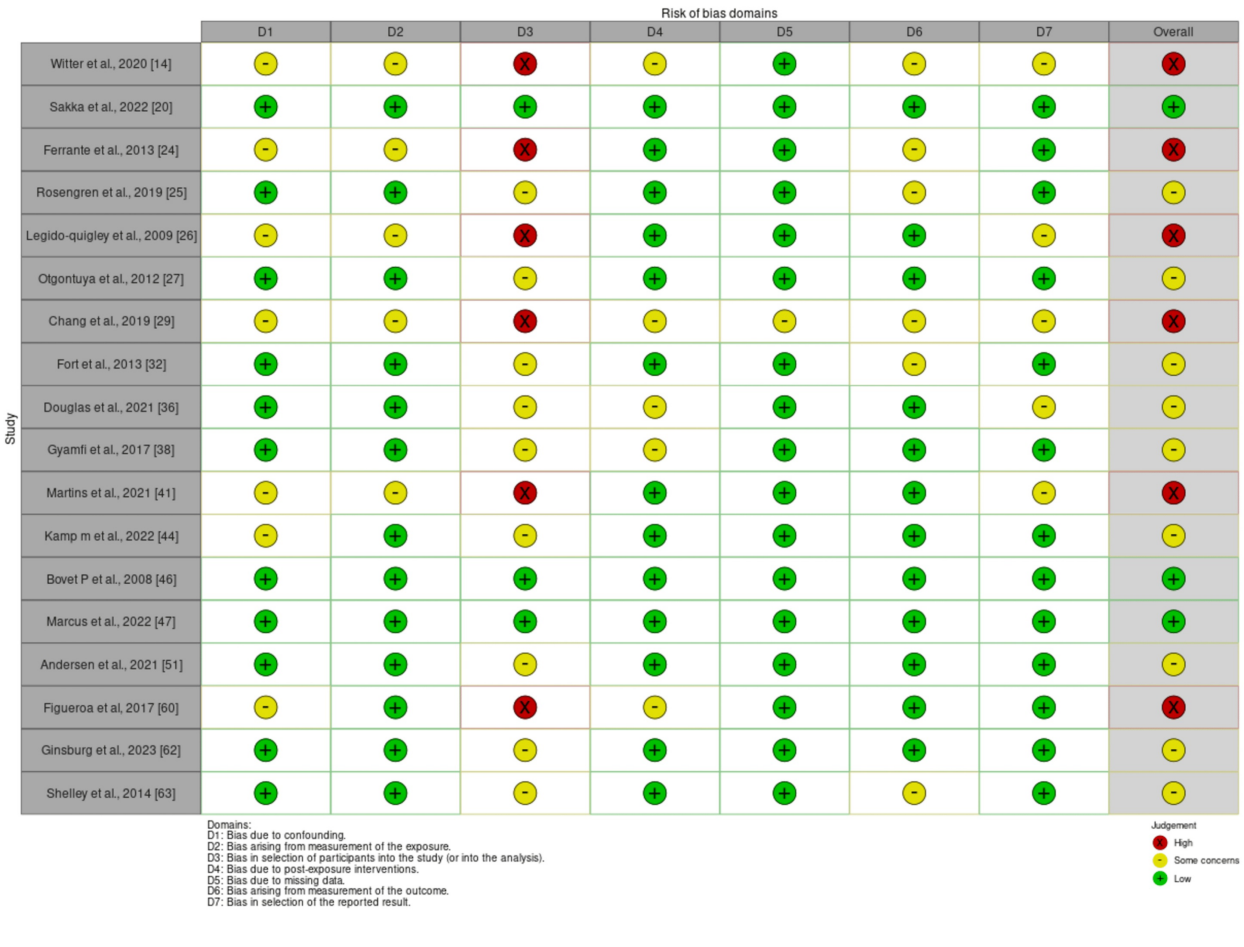
The ROBINS-E (Risk Of Bias In Non-randomized Studies - of Exposure) tool.

**Figure 3:**
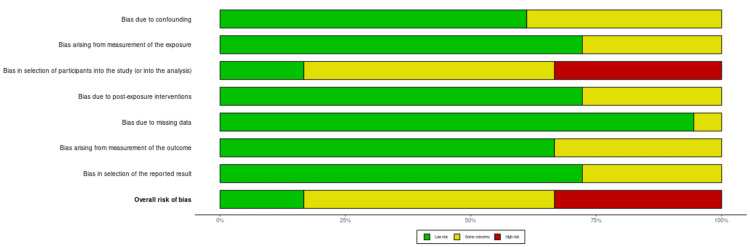
Summary plot for the included studies showing the risk of bias assessment.

We performed a risk of bias assessment of the selected studies using the ROBINS-E (Risk Of Bias In Non-randomized Studies - of Exposure) risk of bias appraisal tool. Both the traffic light plot and summary plot for the included studies are provided below in Figures 2, 3.

Results

Finally, 18 studies were analysed. Three were from South Africa, and one each from Argentina, Sri Lanka, Jordan, Vietnam, Tanzania, Ghana, Sahrawi, Mongolia, Cambodia, Ghana, and Sierra Leona. Four multi-country studies used primary data from national surveys to analyse the barriers affecting the primary prevention of CVDs. These four studies were included because they provided deeper insights and country comparisons in LMICs. The Prospective Urban Rural Epidemiology (PURE) study focused on socioeconomic status and risk of CVD in a mix of HICs and LMICs [25]. Of the 18 papers included in the final review, 10 were purely quantitative, three were mixed qualitative and quantitative methodologies, and five were purely qualitative.

Quality of Studies

The quality of the included studies was generally good. Qualitative studies had a clear description of the methodology; some described the sampling frame, be it purposive or convenience sampling. Focus group discussions were a key pathway to elicit responses from participants and draw well-coded themes that were analysed using computer software, such as Atlas.ti® (ATLAS.ti Scientific Software Development GmbH, Berlin, Germany), as well as the study's challenges and shortcomings. However, in this review, the qualitative studies generally had small sample sizes. In the study, “Patients' experiences on accessing health care services for management of hypertension in rural Bangladesh, Pakistan and Sri Lanka: A qualitative study”, conducted in three countries, i.e., Sri Lanka, Bangladesh, and Pakistan, only 60 participants were contacted. The authors remarked that more participants were included in the study [26]. Some studies used a mixed methodology, collecting quantitative data, and then reinforcing the results with qualitative data in two different phases.

Barriers to Primary Prevention of CVDs

After reviewing all 18 studies, certain themes began to form. The barriers were divided into three main categories, including patient, physician, and health system barriers, which pose significant impediments to accessing care. Barriers led to diminished willingness to access care and a lack of a conducive environment for exchanging information or patient education between patients and healthcare providers. Barriers create unappealing and poor health infrastructures.

Patients with chronic conditions need lifelong follow-up, which causes an emotional toll on the patients; they sometimes wait for a long time before seeking treatment [27]. Patients adjust to the disease state by employing coping mechanisms such as divine help, family support, trust in physicians and medical systems, changing their lifestyles, and being more self-conscious [28]. Stress from illnesses such as diabetes triggers long-term CVD.

Patient barriers

Three studies that discussed barriers from the patient’s perspective were identified. One was a multi-country study conducted among 60 patients in three countries: Sri Lanka, Bangladesh, and Pakistan. This qualitative study involved in-depth interviews. Another study conducted in Uganda examined the challenges of diabetes and hypertension in rural Uganda [29]. Finally, a study conducted in Mexico and Costa Rica investigated barriers and facilitating factors for disease self-management; this qualitative analysis examined the perceptions of patients receiving care for type 2 diabetes and/or hypertension in San José, Costa Rica, and Tuxtla Gutiérrez, Mexico.

The study conducted in Bangladesh, Pakistan, and Sri Lanka is a small study with approximately 20 participants per country. However, it has a robust methodology in terms of the study design and analysis. Participants were purposively selected to ensure information-rich sources from previous study databases. This study was part of another cluster-randomized controlled study evaluating a multicomponent intervention. The intervention included home health education, government community workers, blood pressure monitoring and referral, training of both private and public health workers, and an incentivized performance-based approach to lowering blood pressure.

These three countries have a high prevalence of hypertension, especially in Pakistan, where an estimated 40% of the inhabitants have high blood pressure [30]. The study utilized the Levesque framework, which includes approachability, acceptability, availability/accommodation, affordability, and appropriateness [3].

Inadequate Information From Healthcare Providers

One of the emergent barriers in these studies was the insufficient information provided by healthcare providers, impacting their engagement with both physicians and patients. A lack of adequate knowledge about the disease reduces the engagement with healthcare providers [31]. For instance, in the Sri Lankan study, participants reported a lack of advice from doctors regarding physical activity and diet to control hypertension [26].

In the Ugandan study, most of the information on the disease came not from doctors but from laypersons in the community and social circles. Participants also criticized healthcare providers for not providing accurate information to patients, stating, “The doctor just gives the drugs; I don’t know their names”. These findings were confirmed by interviews with healthcare providers, revealing that they are handicapped due to a lack of resources for public education [29]. In Mexico and Costa Rica, a study using a transtheoretical method explored the barriers and facilitating factors for disease self-management. The qualitative analysis of perceptions among patients receiving care for type 2 diabetes and/or hypertension revealed complaints about the inadequate information provided by healthcare providers [32]. The transtheoretical model suggests that behaviour change occurs over time through six stages: pre-contemplation, contemplation, preparation, action, maintenance, and termination. This can lead to self-efficacy and increased patient knowledge, facilitated by information from healthcare workers [32].

Unfortunately, patients needing weight loss or smoking cessation were not provided any information on these parameters. It was highlighted by participants that they were routinely prescribed pills and had no idea when to stop taking them. According to Levesque et al., the inability to engage is a key to accessing healthcare [3]. Self-advocacy enables patients to voice their concerns, address their unmet needs, and maintain control over their lives [33].

Lack of Symptoms and CVD as a Silent Phenomenon

Another barrier from the patient’s perspective is the asymptomatic nature of CVDs at the early stages. As a result, patients tend to downplay their illness until it becomes complicated. Hypertension is often referred to as a silent killer, because initially, it does not show symptoms, but progressively causes organ damage [34]. In a qualitative study conducted in Sri Lanka, Bangladesh, and Pakistan, some participants reported that they had experienced the symptoms but lacked specific information on the disease [26]. On the other hand, most participants reported a lack of awareness about the asymptomatic nature of hypertension and its serious consequences. The few who were aware of the consequences described them as “dramatic”, such as neck tearing [26]. In the Ugandan study, some patients spent almost a year thinking about their disease before seeking medical care [29].

Delays in Screening Can Lead to Delayed Diagnosis

In the Cambodia and Mongolia STEPS (STEPwise approach to surveillance) survey analysis, it was reported that 50% of the participants in Cambodia had previously documented blood pressure checks. In Mongolia, this number was 65.8%, indicating better screening coverage. However, nearly 50% of the diagnosed patients in both countries remained untreated due to healthcare shortages. A similar proportion of patients with diabetes in need of treatment remained untreated. The control of blood pressure was approximately 25%, compared to 15% for blood sugar control. At the time of the surveys, no formal guidelines for universal, targeted, or opportunistic screening for CVD risk factors existed in either country. However, both Cambodia and Mongolia prioritize the early detection of NCDs in their current national policies and program documents [27].

Health Financing Barriers

The lack of insurance and out-of-pocket costs were mentioned as barriers to accessing primary prevention of CVDs. In the Ugandan study, the government supported health care in the public health sector. However, there were frequent drug shortages in public facilities, and patients could only access private pharmacies at a substantial cost. Out-of-pocket spending can be as high as 40% of the health budget in LMICs, presenting a significant barrier to accessing care [35].

Financial hardships were also reported in a Mexican study, especially in Chiapas, where patients could not receive timely medication from health centres and had to purchase them from the private sector. Financial concerns were expressed in various ways, such as opportunity cost, inability to work because of illness, expenses for medication and medical examination, higher costs of prescribed healthy diets, and the caregiver's cost [32]. In Pakistan, the level of insurance is low, and the majority reported that they had no form of health insurance. Patients found it difficult to make ends meet, especially with their medications for chronic conditions [26].

It was very difficult for patients to make both ends meet with this health problem. This financial strain was not solely due to medication costs but also included other service fees, such as arranging an ambulance, purchasing medications, undergoing investigations, and the doctor’s fee [26]. In some instances, medicine was provided free of charge by the government, greatly easing the financial burden on patients [26].

Physician barriers

Staff Shortages

Several studies have specifically examined the barriers faced by physicians in delivering primary health care to patients. One major concern is the severe shortage of healthcare workers in LMICs. According to the World Bank report in 2020, the patient-doctor ratio in many LMICs is 1:10,000. In Kenya, the ratio is 2:10,000 patients. This low ratio has also been reflected in qualitative studies conducted in several countries. In the Limpopo study, it was found that there was a shortage of nurses and other healthcare workers to handle NCD patients, along with poor accessibility to health facilities [36]. Those who live through natural attrition such as retirement or death are not replaced. They also mentioned the lack of resident doctors and the fact that patients had to be referred to another facility, i.e., Mankweng Hospital. Support staff, such as administrative officers handling paperwork and filing, were unavailable. The health workers were overstretched, as reported by one nurse who complained of covering several wards at a time, and the facility had limited opening hours and was closed at night. If an incident occurs at night, the patient must be referred to another centre. This limits access to the clinic, as described in detail in the Levesque model. The use of modern technology, such as computers, is quite limited, and there is an opportunity to modernize and use currently available capabilities to improve the management of CVDs [36].

It was observed that natural attrition, such as retirement or death of healthcare workers, was not followed by replacements. Participants also highlighted the absence of resident doctors, necessitating patient referrals to another facility. Support staff, including administrative officers for paperwork, were reported as unavailable. Nurses expressed being overstretched, covering multiple wards simultaneously. Clinics had limited operating hours, closing at night, and emergencies during nighttime required patients to be referred elsewhere, further limiting clinic accessibility. These operational constraints are detailed in the Levesque model. The use of modern technology, such as computers, was quite limited, presenting an opportunity for modernization to enhance capabilities for improving CVD management [36].

In response to staff shortages, some countries have implemented task shifting as an innovation. Task shifting is an established concept promoted by the World Health Organization to expand the scope of clinical activities for nurses and lower cadres in the health workforce. This approach aims to optimize human resource utilization by delegating clinical duties to health workers with shorter training durations, thereby maximizing available human resources [37].

In Ghana, the Task Shifting Strategy for Hypertension Control (TASSH) was implemented in 2012 through a mixed quantitative study, followed by a qualitative study [38]. The quantitative study aimed to identify gaps in hypertension management and design targeted interventions. In Ghana, nurses typically receive minimal training in CVD management. Enrolled nurses received basic on-the-job training in NCDs, while community nurses primarily focused on reproductive health and family planning. Upon enrolment in the study, nurses expanded their roles to include tasks such as blood sugar and cholesterol checks, as well as risk assessment using WHO scores [38].

In Uganda, the task-shifting approach to village health teams (VHTs) encountered scepticism from both patients and healthcare providers. VHTs consist of community health workers primarily involved in HIV care and treatment, but they have not been extensively utilized for preventing or managing CVDs. Their main role has been to refer patients with NCDs to the nearest clinic. Participants reported that VHTs were not assigned the role of managing diseases like hypertension (HTN) and diabetes mellitus (DM), leading them to refer all such cases to hospitals. Both patients and healthcare providers expressed doubts about VHTs' capability to manage diabetes and hypertension, emphasizing that infectious diseases are perceived as simpler to understand compared to the complexities of chronic conditions like diabetes and hypertension [29].

Participants claimed they lacked clarity about the roles of VHTs, believing that managing conditions like HTN and DM was too complicated for them. They felt VHTs were only capable of handling simpler diseases like malaria and diarrhoea. Consequently, many villagers with chronic conditions had minimal interaction with VHT members due to their limited knowledge about HTN and DM [29].

In contrast, patients in Bangkok, Pakistan, and Sri Lanka have advocated for greater involvement of community health workers in managing hypertension. There are suggestions that community health workers should be equipped with manometers to enhance their effectiveness [26].

Absence of National Guidelines

The national guidelines for treating NCDs are not universally available. In a South African study, the availability and adherence to CVD guidelines were noted as facilitating access to primary prevention of CVDs [36]. Some guidelines and protocols provide information on initiating treatment, managing, and preventing diabetes, along with a well-defined essential drug list. However, in Argentina, participants lamented the absence of guidelines in health facilities. Another identified barrier was the difficulty in obtaining information in an accessible format. Adherence to guidelines was seen as potentially limiting independent decision-making, with many physicians preferring autonomous approaches [39]. Social workers faced similar challenges, overwhelmed by urgent social and health issues that hindered their ability to prioritize prevention activities.

In Sierra Leone, primary care lacks specific guidelines, relying heavily on healthcare providers' knowledge. This is exacerbated by a weak referral system and a shortage of specialist doctors [14].

Absence of Shared Decision-Making

Shared decision-making is a new concept empowering patients to engage in dialogue with healthcare providers about their health management. This model emphasizes patient-centred care in clinical practice [40]. The NHS in England extensively adopts this collaborative approach, where clinicians partner with patients to jointly decide on treatment plans [40]. Key components of shared decision-making include personalized care and support planning, facilitating informed choices, legal rights to choose, social prescribing, community support, supported self-management, personal health budgets, and integrated personal budgets [40].

In a Mexican study, significant vertical communication was noted between physicians and patients regarding barriers and facilitators of self-management [32]. In Jordan, a descriptive randomized cross-sectional survey involving 157 pharmacists was conducted from April to August 2018 [41]. Jordan experienced a high prevalence of smoking among adults (70%) and 34% among 13-to-15-year-olds, with water pipe use prevalent among 30% of university students. The study aimed to assess pharmacists' knowledge, attitudes, and practices in smoking cessation services. It revealed that nearly half of the pharmacists had a negative attitude towards smoking cessation, believing it futile if patients were unwilling to quit. A quarter of pharmacists viewed smoking cessation counselling as ineffective [41].

Almost all pharmacists (93%) reported lacking or never providing educational material on smoking cessation (SC). They expressed a lack of motivation due to the absence of reimbursement or compensation for these services. A total of 70% felt strongly that there was insufficient economic reward for SC services. Pharmacists emphasized the need for additional training to enhance the effectiveness of SC services, noting that many patients were too rushed to benefit from counselling. Those with a Pharm D or Bachelor of Pharmacy degree showed a more negative attitude towards counselling patients. Interestingly, pharmacists who smoked themselves were less willing to engage in SC services. The study also found that only 24.6% of pharmacists routinely asked patients about their smoking status, leaving many patients unreached [41].

In Pakistan, Martins et al. established that factors such as family support, motivation, concern for health, disease self-awareness, and marriage were significantly associated with the willingness to quit smoking [41]. The study found that 74% of former smokers believed that quitting would improve their health, highlighting the need for increased education of smokers. However, only 13% of smokers quit following prompts by doctors [42].

Traditional healers provide alternative medicine to patients, which is a barrier to evidence-based practice for CVDs. For example, in Bangladesh, traditional and Ayurvedic medicine have been identified as obstacles to the primary prevention of CVDs. Despite this, some patients still opted for allopathic medicine for their ailments [26].

In South Africa, there is a strong following of traditional healers. The formal healthcare system perceives their role as facilitators rather than barriers to CVD prevention. The healthcare system values its relationship with traditional leaders, often consulting them first when problems arise. Traditional leaders hold regular meetings, where health issues are discussed, concerns are shared, and collaborative solutions are found to address CVD-related problems. This involvement of traditional leaders is seen as a good strategy for improving health outcomes [36].

In Uganda, some patients first try traditional medicine and only go to the hospital as a last resort once they have exhausted herbal medicine options. A closer relationship between the formal health system and traditional medicine may be required to facilitate early diagnosis and treatment of CVD in some communities.

In Sierra Leone, traditional healers have been accused of overstepping their boundaries. Despite this, many NCD patients trust traditional healers due to their lower costs and shared community beliefs. Some government facilities and district health management teams in Makena have proposed inviting traditional healers to participate in their routine meetings [14].

Religious beliefs can also impact disease management. In a Mexican study, faith was identified as a facilitator of disease management, with participants relying on prayer to live longer. However, sometimes religious activities can lead patients into denial about their condition.

Health system barriers

The healthcare systems in many LMICs struggle to address chronic CVDs due to various deficiencies. Challenges include inadequate scope and personnel, poor infrastructure, ill-equipped laboratories, overworked staff, and limited budgetary allocations from central governments [38]. Moreover, many LMICs fail to allocate the recommended 15% of their budgets to healthcare, as advocated by the WHO and regional economic bodies [42,43]. Brain drain is a significant issue, with healthcare professionals often leaving for better opportunities in Europe and the US due to insufficient resources and poor working conditions at home. South Africa, despite being one of Africa's most advanced economies, faces similar challenges in healthcare delivery and medical advancements.

A study was conducted to explore clinicians' perceptions of precision medicine and risk stratification through genomic incorporation. While most clinicians believe the role of genomic testing in risk assessment, only one-third have received sufficient training. Patients also have limited genetic literacy, posing challenges for clinicians to advance genomic approaches. Making guidelines for genomic testing can enhance its usage in clinical practice [44].

Sierra Leone, a country that has faced prolonged conflict, encounters unique challenges in its healthcare system. Many health departments rely on donor funding, prioritizing projects such as HIV, malaria, and family planning. Like other countries in Sub-Saharan Africa, Sierra Leone has weak health systems, inadequate resources, and struggles to address both communicable diseases and NCDs. There is a notable absence of national-level funding and programs for NCDs and there are no efforts to improve health literacy about NCDs and CVDs [1,14].

Sierra Leone's healthcare efforts have largely focused on maternal health and communicable diseases among children. The country faces challenges with human resources, with a doctor-patient ratio currently at 1:10,000 (World Bank SL, 2023). This shortage impacts the delivery of care, particularly for NCD treatments, which are not reimbursed and fall under a costly drug recovery program. The last STEP survey conducted in 2009 shows budget shortages for NCDs, leaving the healthcare system under-resourced and understaffed [14].

Adherence to Medication

**Figure 4:**
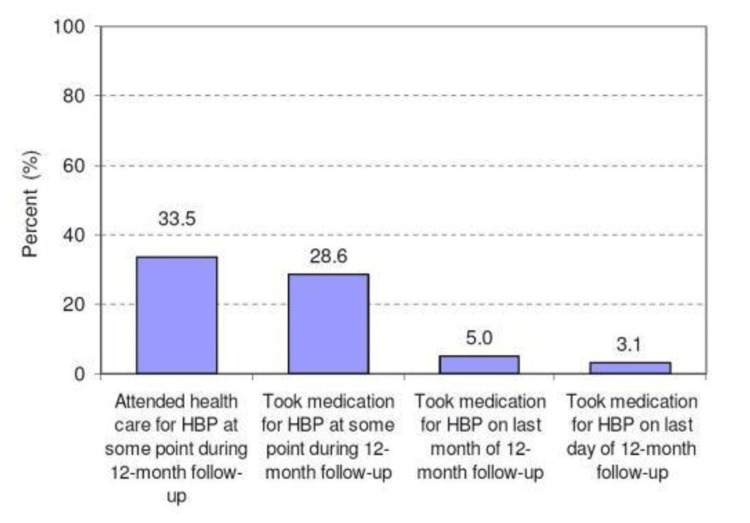
Proportion of all untreated hypertensive patients at baseline who attended health services for hypertensive therapy following diagnosis after the initial screening for hypertension. HBP: high blood pressure. Figure reproduced from Bovet et al. [46]. Permission was obtained from the authors to reproduce the figure.

CVDs require long-term medication, potentially for life. Patients must receive regular counselling about the nature of treatment. Proper education is necessary regarding the importance of medication adherence. According to the World Health Organization (WHO) report, there are five multidimensional models of adherence, including patient-related factors such as self-efficacy, perceived health, beliefs about efficacy, and knowledge of medication [45]. These factors include social and economic factors, limited language proficiency, low health literacy, unstable living conditions, provider-patient and healthcare system factors, knowledge about the disease, perceived risk, perceived benefits, condition-related factors, therapy-related factors, duration of therapy, complexity of medication, patient-related factors, and restricted formularies [45]. In a Tanzanian quantitative study titled, "Low utilization of health care services following screening for hypertension in Dar es Salaam (Tanzania): a prospective population-based study," a prospective population-based approach was used. The study observed treatment adherence among patients with high blood pressure. Significant attrition was noted from the study's onset to its endpoint, with the rate of antihypertensive treatment decreasing from an estimated 34% at the beginning to 3% by the end of the 12-month period. Despite assumptions made regarding adherence, there was a significant decline observed over time. Figure 4 illustrates the data depicting the low utilization of healthcare services following screening for hypertension in Dar es Salaam, Tanzania [46].

Lack of Medication and Equipment

Several studies, both quantitative and qualitative, have cited the lack of drugs at healthcare facilities as a significant barrier. This issue has been reported in studies conducted in Uganda and Sri Lanka. In South Africa, a qualitative study with primary healthcare managers highlighted "shortages of medical supplies and equipment" as significant issues. Shortages of medications were also reported, leading providers to sometimes be unable to provide all prescribed medications. Additionally, shortages of equipment such as blood pressure machines and glucometers were noted, forcing reliance solely on history-taking in some cases [36].

The role of statins in the primary prevention of CVDs has been well-documented over the past several years. Statins are widely recommended for preventing CVD. However, their utilization remains very low. According to the WHO, only 50% of eligible patients receive treatment. This target has not been achieved by the vast majority of LMICs for primary prevention of CVD. In the Lancet review involving 41 countries in LMICs, data from the WHO STEPS survey showed that statin use for primary prevention was 8%, while it was 21% for secondary prevention [47]. This sharply contrasts with the levels in upper-middle-income countries, where statin use for primary prevention is seven times higher than in LMICs. The review indicated that no country achieved WHO goals for primary prevention, highlighting an urgent need for scaling up efforts. Challenges such as higher costs of screening and medications, along with the non-inclusion of statins in essential medical drug lists, further hinder their widespread use [47].

According to the Prospective Urban Rural Epidemiologic (PURE) study, based on the socioeconomic status and CVD risk in 20 low-, middle-, and high-income countries, statins could cost up to 17% of discretionary income in urban areas and even higher, up to 49%, in rural areas, thus creating a significant financial barrier to their utilization [48]. In the PURE study, lower education was identified as a significant barrier to accessing health care in LMICs. Conversely, higher education and wealth in LMICs were associated with better health outcomes [48]. However, STEPS surveys have inherent weaknesses such as recall bias and are not universally conducted across all LMICs due to limited resources. Large LMICs with substantial burdens of CVDs, like India and China, were excluded from these surveys.

Poor Infrastructure and Lack of Green Spaces

In several studies, lack of infrastructure in communities and long distances travelled by patients to health facilities were cited as barriers to accessing primary prevention of CVDs. In a South African study known as the Limpopo study, it was reported that health infrastructure was inadequate to accommodate patients with CVD. There was a lack of space to perform procedures, and privacy for patient examinations was also insufficient. These infrastructure deficiencies significantly hinder efficient health service delivery for patients with chronic conditions attending primary care services [36].

The WHO recommends that adults perform 150-300 minutes of moderate-intensity aerobic activity per week, accompanied by moderate or greater-intensity muscle-strengthening activities involving major muscle groups. A quantitative study conducted among Sahrawi refugees in protracted confinement at the Algerian border reported limited physical activity among the refugees. Physical activity was defined as more than half an hour of daily activity, and approximately 43% of the participants reported lower physical activity levels than recommended. In this study, women reported less physical activity and were less mobile than their male counterparts. Subjectively, 28% of the women believed their physical activity was low, while 66% were satisfied with their physical activity levels. Among men, 23% reported their physical activity was low or far too low, with only 14% believing it was slightly or far too high. Women also reported significantly less walking per week compared to men. The study concluded that almost half of the participants did not reach adequate physical activity levels, with older males over 60 years and those with higher education more likely to have lower physical activity levels [49].

Fragmented or Absent Data

Health information management systems in LMICs have not been fully deployed, which limits the country's ability to have a comprehensive dashboard of its overall health status. In Sierra Leone, data from the STEPS survey were incomplete, with significant gaps and missing chunks of data. In many countries, health data are predominantly paper-based and stored in files. In Argentina, doctors expressed frustration over the absence of proper information technology systems to streamline their workload. Poorly organized follow-up visits and a lack of risk stratification tools to aid in disease management were reported [39].

In the Limpopo study, healthcare workers were not proficient in using information and communications technology (ICT) equipment for electronic medical records, resulting in continued reliance on paper-based patient files. The healthcare facility's infrastructure was reported to be too small, lacking adequate privacy for patients during vital sign assessments and interviews due to the absence of dedicated rooms for these procedures. Vital signs were taken where patient files were stored, further reducing available space and potentially leading to inaccuracies in reporting, thereby affecting patient management. According to the NHS guidelines, patients should be in a relaxed, comfortable position, preferably in a quiet room, to ensure the accuracy of vital sign readings [50].

Discussion

LICs and LMICs face many challenges in the management of CVD. Many countries do not prioritize NCDs, including CVDs, in their national health plans. LICs have the lowest NCD implementation plans [51]. For a long time, the focus has been on infectious diseases, such as malaria and HIV, which receive the bulk of donor support from LICs [51,52]. The prevalence of NCD risk factors continues to increase. Between 2003 and 2007, the risk factors were 14.8%. This has rapidly increased to 44% between 2013 and 2017. Lifestyle changes and increased awareness have led to a substantial increase in the prevalence of CVDs. This review examined 18 papers with original research on qualitative and quantitative studies. Barriers to accessing the primary prevention of CVD can be divided into three sections: physician factors, patient factors, and health system barriers. Unfortunately, little research has been conducted on LICs. Approximately 90% of the studies were excluded because they originated from HICs. Among the few studies that fit the inclusion criteria for the review, barriers to the primary prevention of CVDs are similar in most LMICs. There is a need to focus more on primary prevention and allocate more resources at primary healthcare facilities to initiate programs geared towards health promotion. For example, in Sierra Leone, no funds are available to prevent CVDs. Healthcare providers do not spend time providing information about lifestyle changes or non-drug interventions, even in more advanced countries like Argentina [53].

Despite their higher population densities, LICs have very poor health ecosystems and few healthcare providers. There are more doctors per capita in the USA than in countries like Kenya, where one doctor serves 10,000 people. This strain on human resources could be due to many factors, including brain drains. In Nigeria, before the COVID-19 pandemic, 88% of Nigerian doctors and 50% of nurses considered working abroad due to poor working conditions at home [54]. According to Misau et al., brain drains negatively affect health delivery in LICs [55]. The physician-to-population ratio in Africa is 1/100,000, while in the USA, it is 280/100000. Of the health workforce in the USA, 25% are foreign-trained. In contrast, in a low-income country such as Lesotho, there is one doctor per 10,000 inhabitants despite the country having a very high HIV prevalence of 28% [56].

LMICs have adopted a famous task-shifting strategy to mitigate the shortage of skilled workers, especially at the primary care level. Many countries have deployed community health workers across LMICs. Uganda, for example, has VHTs that have focused on HIV and maternal health for a long time. Could they be deployed to cover CVDs? This is possible if they are trained and provided with the necessary equipment for measuring blood pressure. In Ghana, nurses are trained in primary cardiovascular prevention, diagnosis, and treatment of patients in the community. Research has shown that, with proper training, they can initiate treatment at the primary care level [38].

CVDs are a subtle disease and are often not dramatic unless they are complicated. The onset of symptoms is early and insidious. This makes many patients unaware of the urgency of treatment, and many years pass before they act. Patients have also been reported to delay treatment with a wait-and-see approach, afraid of becoming overly dependent on drugs [29]. In a review of patients with acute myocardial infarction in Iran, it was found that this could be due to various reasons, including wishing the symptoms away, hoping the symptoms would resolve spontaneously, or simply disregarding them [57]. CVD care is also expensive for diagnosis. Cholesterol tests are prohibitive, yet they are a gateway to access treatment for atherosclerotic cardiovascular disease (ASCVD) [47].

Physical barriers, such as long distances to health centres, which make them less accessible, also hinder healthcare access. A study conducted in Tanzania found that long distances were often a barrier to health access [58]. Children who lived more than 5 km from health centres had a 17% increase in mortality compared to those who lived less than 5 km from the centres [58]. Moreover, health facilities do not open for 24 hours, and limiting the opening hours makes them less accessible, especially when patients consider the opportunity costs of visiting the doctor.

Poor infrastructure at health facilities, overcrowding, and limited space for taking patients' vitals could create discomfort and dissuade asymptomatic patients from seeking health care [36]. Lack of transportation has been cited as a barrier to accessing primary care in Ghana. In Pakistan, physician barriers, such as a small number of healthcare personnel, lack of adequate training in the management of CVDs, or absence of guidelines, were mentioned as hindrances to accessing primary prevention of CVD [44]. In countries such as Argentina, there are no guidelines, and physicians use their skills to prevent and treat CVD. However, in South Africa, guidelines are available for managing CVD. It was also reported that many healthcare workers did not offer patient counselling to prevent CVDs. Healthcare providers were overwhelmed by work and developed a top-down approach when addressing patients [44]. The lack of shared decision-making pathways contrasts with what happens in HICs, such as in the UK, where a patient is offered detailed information on the treatment plan and should participate in the decision-making process.

Weak health systems are a characteristic of LMICs. Thus, infrastructure may not be adequate. A shortage or absence of drugs, equipment, and medical diagnostics can lead to delays in diagnosis and treatment initiation. In Sierra Leone, NCD drugs are not reimbursable and must be paid out-of-pocket. However, this severely limits their availability. Low-quality generic drugs are often found at LMICs.

Finally, cultural, religious, and traditional factors have also been featured prominently in this review. In South Africa, obesity is considered a positive attribute [59]. Children are considered healthy, and childhood obesity reverses [59]. South Africa has one of the highest prevalence rates of obesity. This is projected to increase to 47.7% in females and 23% in males by 2025 [60]. Obesity is also associated with contentment. It reflects wealth. Obesity is a precursor of type 2 diabetes and a major risk factor for CVD. Perceptions of weight by individuals are instrumental in shaping intervention programmes.

Traditional healers and alternative medicines have long occupied space in the healthcare arena [61]. In South Africa, they have been formally incorporated into the healthcare system in some districts because of their high social capital. The Traditional Healing Act of 2007 in South Africa clearly defined the role and scope of this important segment of healthcare providers. It also provides a regulatory framework for the training and registration of traditional healers. Their practice includes spiritual, counselling, cultural, herbal, and referral of patients to the local clinics [61].

In Sierra Leone, they have been accused of overstepping their mandates by going beyond what they should ordinarily offer [14]. In Bangladesh, the use of Ayurvedic and allopathic medicines is still in practice [26]. Traditional healers can form an integral part of the healthcare system, and efforts should be made to tap into their circles and create a symbiotic relationship to reduce barriers to the primary prevention of CVDs. The results are displayed in Table 2.

**Table 2:** Results of various studies included in this systematic review. NCD: non-communicable disease; SRH: sexual and reproductive health and research; MCH: maternal and child health; HCP: healthcare personnel; LIC: low-income countries; CVD: cardiovascular disease; VHT: village health team; FGDs: focus group discussions; PHC: primary health care; LMICs: low and middle-income countries; IPAQ-SF: International Physical Activity Questionnaire - Short Form.

Study title	Author name	Country of publication	Study type	Quality	Barriers
Opportunities and challenges for delivering non-communicable disease management and services in fragile and post-conflict settings: perceptions of policy-makers and health providers in Sierra Leone	Witter et al. (2020) [14]	Multiple	A qualitative study with a high risk of bias: small sample from only 2 out of 16 districts. Does not address how conflict acts as a barrier to access. Results are overly generalized. Recruitment bias through purposive and convenience sampling.	Small sample size, non-randomized selection targeting those interested in NCDs. Research teams relied on local leaders to direct them to potential interviewees. Group modelling sessions were used.	Funding, conflicts, political commitment, low focus on NCDs, NCDs not prioritized; instead, MCH and SRH receive more support. Lack of community empowerment, limited opening hours in public facilities, lack of national guidelines, limited primary care services, reliance on traditional healers, lack of medicines in public facilities, NCD medicines only available for purchase, basic diagnostic equipment not in use, poor quality drugs, incidents of fraud, and staff involvement in buying and selling medicines.
Knowledge, attitude, practice and perceived barriers towards smoking cessation services among community pharmacists	Sakka et al. (2022) [20]	Jordan	Quantitative	Cross-sectional survey using self-administered questionnaires and randomized sampling from the Jordanian pharmacy register.	Perceived as a waste of time and not reimbursable; 74% believe patients should quit independently. One-third have never assessed a patient’s willingness to stop, while 93% have provided patient information on smoking cessation.
Barriers to prevention of cardiovascular disease in primary care settings in Argentina	Ferrante et al. (2013) [24].	Argentina	Tailored intervention, mixed qualitative and quantitative methods with baseline and post-intervention analysis.	Robust, including before and after analysis, tailored intervention.	Lack of knowledge of guidelines, lack of guidelines, guidelines as an impediment to independent thinking, workload, lack of motivation, lack of knowledge sharing among HCPs, poor follow-up visits, manual records, no risk stratification, and lack of adequate infrastructure at the PHC level.
Socioeconomic status and risk of cardiovascular disease in 20 low-income, middle-income, and high-income countries: the Prospective Urban Rural Epidemiologic (PURE) study	Rosengren et al. (2019) [25]	Multiple	Quantitative study with a high risk of bias towards India, with many low-income countries (LICs) either left out or contributing fewer numbers. India and Bangladesh represented 88% of LIC participants.	Prospective study, large sample size of 182,000 participants, follow-up of 7.5 years, 20 countries. Systematic data collection.	Education is a barrier to accessing care, across all the countries. Health system barriers.
Patients' experiences on accessing health care services for management of hypertension in rural Bangladesh, Pakistan and Sri Lanka: A qualitative study	Legido-Quigley et al. (2009) [26]	Sri Lanka	Qualitative	Robust methodology, randomization, training of interviewers, coding, interviews conducted with respondents in their homes, and privacy guaranteed.	Lack of awareness, absence of symptoms, preference for alternative medicine and Ayurvedic treatments, cultural and religious factors, lack of information from healthcare providers, long waiting times, medication shortages, long travel distances, out-of-pocket expenses, competing interests, travel fare, food costs, inability to make ends meet, fast but expensive private facilities, patients only mentioning illnesses they can afford to pay for, and patients seeking information.
Individual-based primary prevention of cardiovascular disease in Cambodia and Mongolia: early identification and management of hypertension and diabetes mellitus	Otgontuya et al. (2012) [27]	Mongolia/Cambodia	Quantitative	Step survey, cross-sectional study with self-reporting bias.	Screening gaps, lack of diagnosis, treatment gaps, absence of global CVD risk assessment, inadequate national guidelines, insufficient patient education, smokers not receiving smoking cessation advice, and obese individuals not being advised to reduce weight.
Challenges to hypertension and diabetes management in rural Uganda: a qualitative study with patients, village health team members, and health care professionals	Chang et al. (2019) [29].	Uganda	Qualitative	Semi-structured face-to-face interviews were conducted with patients, healthcare providers (HCPs), and village health teams (VHTs). Theoretical sampling methodology was employed.	Patient challenges include poor information from healthcare providers, lack of access to medication, exclusion from the medical decision-making process, negative perceptions of healthcare providers, use of herbal medications, delayed referrals, concerns about village health team (VHT) roles, and financial barriers.
Barriers and facilitating factors for disease self-management: A qualitative analysis of perceptions of patients receiving care for type 2 diabetes and/or hypertension in San José, Costa Rica and Tuxtla Gutiérrez, Mexico	Fort et al. (2013) [32]	Mexico	Quantitative	Focus group discussions (FGDs) are useful for obtaining in-depth information on the potential for recruitment bias.	Patient-physician communication, lack of information sharing, depression due to slow progress in weight loss despite efforts, concomitant illnesses like endometriosis complicating the management of NCDs, lack of green spaces, caregiving responsibilities for family members, managing multiple medications and difficulty in keeping track, financial constraints, competing interests related to food, high dependency on others, reliance on faith and God, gender issues particularly for single women, and significant out-of-pocket expenses.
Facilitators and barriers in prevention of cardiovascular disease in Limpopo, South Africa: a qualitative study conducted with primary health care managers	Douglas et al. (2021) [36]	South Africa	Qualitative	Convenience sampling, small sample size, and targeting only PHC managers instead of prescribers.	Lack of guidelines, shortage of staff, infrastructural deficiencies, lack of medicines, lack of health promotion services, poor access to primary health care services, lack of data sharing, inadequate access to facilities, poor doctor-patient relationships, and unwelcoming staff.
Training nurses in task-shifting strategies for the management and control of hypertension in Ghana: a mixed-methods study	Gyamfi et al. (2017) [38]	Ghana	Mixed quantitative and qualitative	Good quality, cluster randomized design, well-structured qualitative interviews, and analysis.	Lack of transportation, lack of insurance, high cost of medicines, inadequate healthcare workers, insufficient equipment, poor follow-up visits, language barriers, lack of patient motivation, and absence of specialized clinics like hypertension clinics.
Factors motivating smoking cessation: a cross-sectional study in a lower-middle-income country	Martins et al. (2021) [41]	Pakistan	Quantitative	Recall bias, weak definition of quitting (one week is too short).	Lack of smoking cessation centres and no prompting by doctors to quit smoking.
Clinicians’ perceptions towards precision medicine tools for cardiovascular disease risk stratification in South Africa	Kamp et al. (2022) [44]	South Africa	Quantitative	Small sample size, not representative of primary healthcare physicians.	Poor knowledge of genetics, inadequate risk scoring, lack of knowledge, high costs, lack of guidelines, insufficient funding, shortage of human resources, limited training in precision medicine, and limited time for patient education. Additionally, low genetic literacy is a significant concern.
Low utilization of health care services following screening for hypertension in Dar es Salaam (Tanzania): a prospective population-based study	Bovet et al. (2008) [46]	Tanzania	Quantitative population-based prospective cross-sectional survey	Good, but an old study; definition of hypertension based on >160/95 mmHg systolic, which significantly reduced the prevalence.	Low uptake of treatment, poor adherence, ignorance, competing interests, high costs of medication, inadequate training of health personnel, and insufficient infrastructure.
Use of statins for the prevention of cardiovascular disease in 41 low-income and middle-income countries: a cross-sectional study of nationally representative, individual-level data	Marcus et al. (2022) [47]	Multiple	Quantitative	Prospective study, a large sample size of 182,000 participants followed up for 7.5 years across 20 countries. Systematic data collection.	Statin use for primary prevention is at 8%, while for secondary prevention, it is 21%. Lack of guidelines, costs, lack of political will, statins not prioritized, and perception that statins are less urgent compared to treating high blood pressure.
Insufficient physical activity level among Sahrawi adults living in a protracted refugee setting	Andersen et al. (2021) [51]	Multiple	Quantitative study using the IPAQ-SF (International Physical Activity Questionnaire - Short Form)	Cross-sectional survey using 3-stage cluster sampling, with a high number of dropouts due to work obligations.	Culture, sedentary lifestyle, confinement, harsh environment, behaviour, physical activity not a priority, low education, and high unemployment.
“Culture is so interspersed”: Child-minders’ and health workers’ perceptions of childhood obesity in South Africa	Figueroa et al. (2017) [60]	South Africa	Mixed quantitative and qualitative	FGDs and semi-structured interviews were conducted with a small sample size of 21 participants. Two co-authors independently coded the results during analysis to reduce the risk of bias.	At the cell level, hereditary and biological factors were considered risk factors. At the child level, several factors such as eating habits, food preferences, sedentary habits, screen time, and skipping meals were associated with childhood obesity. At the clan level, factors such as grandparents, force-feeding, parenting styles, and “spoiling” children were associated with childhood obesity. Additionally, child-minder feeding styles, access to junk foods, lack of playground infrastructure, and at the country level, socioeconomic status, food availability, and media were associated with childhood obesity.
A survey of barriers and facilitators to ultrasound use in low- and middle-income countries	Ginsburg et al. (2023) [62]	62 countries	Quantitative	Online survey with a risk of bias: questionnaires were mailed to people known to be using ultrasounds.	Top barriers: access (17%), mentoring and training (14%), education (12%), cost of ultrasound equipment (including gel, probes, and accessories), and competition for use by other departments (38%). Additionally, 96% agree that ultrasound improves the quality of care.
Barriers and facilitators to expanding the role of community health workers to include smoking cessation services in Vietnam: a qualitative analysis	Shelley et al. (2014) [63]	Vietnam	Qualitative	Well-organized focus group discussions using grounded theory for analysis, without randomization.	Inadequate infrastructure, lack of smoking cessation programs and training, and a vertical hierarchical health system.

Recommendation

Most studies on barriers to accessing cardiovascular prevention in primary healthcare have been conducted in more developed countries. Some of the research done on LMICs originated from Western countries; for example, the PURE study and Ginsburg et al.'s study contain data from LMICs, but the principal investigators are based in developed countries [25,62]. There is a big gap in obtaining data from LMICs owing to poor infrastructure and a lack of human resources or electronic health records. LMICs must allocate more resources to health systems to improve cardiovascular research and health delivery. More research is required to develop local parameters and datasets that respond well to the healthcare ecosystem in LMICs.

## Conclusions

Although there is a paucity of literature on the barriers to accessing primary prevention of CVDs, it is encouraging to see some literature from LMICs. Most studies have been conducted in HICs, and the results are superimposed on policies in LMICs. More research must be conducted in LMICs to introduce interventions that incorporate the cultural and religious practices of the Global South. Barriers to primary prevention include weak health systems, lack of health insurance, unavailability of drugs, low numbers of healthcare workers, competing priorities and opportunity costs, and a lack of government policies and financial support.
